# Comments on “Economics of natural disasters and technological innovations in Africa: an empirical evidence” by Okolo, Chukwuemeka et al., DOI (10.1007/s11356-022-22989-8)

**DOI:** 10.1007/s11356-023-30873-2

**Published:** 2023-11-11

**Authors:** Kyoo-Man Ha

**Affiliations:** Faculty of Resilience, Rabdan Academy, Abu Dhabi, UAE

Okolo and Wen (2023) empirically examined how natural hazards (also known as natural disasters) impact technological innovation using a case study in Africa. They extensively analyzed the relationship between the two variables in 45 African countries (between 1990 and 2019) using the quantile regression method (Okolo and Wen 2023). They concluded that natural hazards negatively and significantly affected technological innovation in the region. Natural hazards play a role in decreasing the amount of trade, development of human capital, and use of energy, thus negatively affecting technological innovation.

Two opposing groups have continued to discuss how natural hazards influence technological innovation in emergency management. One group has advocated the negative role of natural hazards in technological innovation, as in the case of Okolo and Wen (2023), Chen et al. (2021), and Haddad and Teixeira (2015). For this group, natural hazards, such as earthquakes, floods, and droughts, generally upset government policies, key infrastructure, and business activities. This leads to the disruption of research plans, decrease in research activities, and reduced research and development (R&D) funds.

The other group has strongly suggested a positive role of natural hazards in technological innovation. These researchers agree that the occurrence of natural hazards has caused both human loss and huge economic damage to human society. Nonetheless, when examining the number of patents or other disaster data in advanced nations, natural hazards eventually spur technological innovation in the region (Miao and Popp 2014). However, the level of positive influence varies across industrial sectors.

Similarly, researchers have maintained that the occurrence of multiple natural hazards has formulated incentives and opportunities for technological innovation in the field (Khitrun 2013). After capital stock decreases due to the physical impacts of natural hazards, regional communities have a strong motivation to replace their deficiencies using innovative technologies. In addition, the impacts of natural hazards have created new opportunities to develop, adopt, and distribute cutting-edge technologies across various regions by increasing R&D expenditures and related programs.

There are at least 18 types of natural hazard, each with unique characteristics; however, they are no longer fundamentally natural (Arosio et al. 2018) because human activities are directly or indirectly involved in their mechanisms. Thus, they have become unnatural hazards. When hurricanes with the same physical power hit two different places, the results are not the same. In addition, risk perceptions and the extent of vulnerability vary among disaster victims. Thus, natural hazards are complex phenomena.

Considering that researchers have faced tremendous difficulties in making a single generalization on research topics due to the complicated aspects of natural hazards, the necessity of case studies has been constantly maintained (Grynszpan et al. 2011). As emergencies are constantly fluid, reliance on case studies plays a significant role in evaluating the relationship between natural hazards and their negative or positive impacts. If rigorously conducted, case studies may produce unique and compelling research results for multiple regions, including Africa.

In addition, heterogeneous research is required to address the need for multidisciplinary information for effective natural hazard management (Shams et al. 2016). Not all studies have examined the same quantity of data on natural hazards; therefore, the results of their research are heterogeneous. When measuring the same data, the results of related research may be inconsistent because of unique research processes or social interactions. Heterogeneous research can be applied to various studies on the impact of natural hazards on technological innovation.

## Data Availability

Not applicable.
